# The 3′-Terminal 55 Nucleotides of Bovine Coronavirus Defective Interfering RNA Harbor *Cis*-Acting Elements Required for Both Negative- and Positive-Strand RNA Synthesis

**DOI:** 10.1371/journal.pone.0098422

**Published:** 2014-05-22

**Authors:** Wei-Yu Liao, Ting-Yung Ke, Hung-Yi Wu

**Affiliations:** Graduate Institute of Veterinary Pathobiology, College of Veterinary Medicine, National Chung-Hsing University, Taichung, Taiwan ROC; Kantonal Hospital St. Gallen, Switzerland

## Abstract

The synthesis of the negative-strand [(−)-strand] complement of the ∼30 kilobase, positive-strand [(+)-strand] coronaviral genome is a necessary early step for genome replication. The identification of *cis*-acting elements required for (−)-strand RNA synthesis in coronaviruses, however, has been hampered due to insufficiencies in the techniques used to detect the (−)-strand RNA species. Here, we employed a method of head-to-tail ligation and real-time quantitative reverse transcription polymerase chain reaction (qRT-PCR) to detect and quantitate the synthesis of bovine coronavirus (BCoV) defective interfering (DI) RNA (−) strands. Furthermore, using the aforementioned techniques along with Northern blot assay, we specifically defined the *cis*-acting RNA elements within the 3′-terminal 55 nucleotides (nts) which function in the synthesis of (−)- or (+)-strand BCoV DI RNA. The major findings are as follows: (i) nts from -5 to -39 within the 3′-terminal 55 nts are the *cis*-acting elements responsible for (−)-strand BCoV DI RNA synthesis, (ii) nts from −3 to −34 within the 3′-terminal 55 nts are *cis*-acting elements required for (+)-strand BCoV DI RNA synthesis, and (iii) the nucleotide species at the 3′-most position (−1) is important, but not critical, for both (−)- and (+)-strand BCoV DI RNA synthesis. These results demonstrate that the 3′-terminal 55 nts in BCoV DI RNA harbor *cis*-acting RNA elements required for both (−)- and (+)-strand DI RNA synthesis and extend our knowledge on the mechanisms of coronavirus replication. The method of head-to-tail ligation and qRT-PCR employed in the study may also be applied to identify other *cis*-acting elements required for (−)-strand RNA synthesis in coronaviruses.

## Introduction

After entry into a cell, the positive-strand [(+)-strand] RNA virus genome first serves as a template for synthesis of viral proteins required for subsequent virus replication. The synthesis of the negative-strand [(−)-strand] complement from the (+)-strand genome is the first step of genome replication and is assumed to be initiated from the 3′ end of the (+)-strand RNA genome. The 3′ end of the synthesized (−)-strand RNA genome is then employed as the site for the initiation of the (+)-strand RNA genome. Therefore, the RNA elements on the 3′ end of (+)- and (−)-strand RNA genome may play critical roles in targeting the viral replication complex to initiate the synthesis of their counterparts.

Bovine coronavirus (BCoV), a (+)-strand RNA virus in the subfamily *Coronavirinae*, the family *Coronaviridae* and the order *Nidovirales*, belongs to the genus *betacoronaviruses*, which includes the mouse hepatitis coronavirus (MHV) [1], [2]. Many *cis*-acting RNA elements in the coronavirus genome required for replication [interpreted as (+)-strand RNA synthesis] have been identified in the past two decades with a full-length infectious cDNA or coronavirus defective interfering (DI) RNA, a surrogate for the coronavirus genome [3], [4], [5], [6], [7], [8], [9], [10], [11], [12], [13], [14], [15], [16], [17], [18]. For *cis*-acting RNA elements in the 3′ UTR of coronavirus, it has been demonstrated that the minimal requirement for replication maps 436 nucleotides (nts) from the 3′ end of MHV DI RNA according to deletion analyses [19], [20]. Within this region, two higher-order structures on the upstream end of the 3′ UTR have been identified as *cis*-acting RNA elements that are essential for coronavirus replication, including a 61-nt bulged stem-loop in MHV [12], [13] and a hairpin-type pseudoknot in BCoV DI RNA [15]. Further downstream of the 3′ UTR is a hypervariable region (HVR) which forms a complex secondary structure. Although this secondary structure was shown to be important for the efficient replication of MHV DI RNA [14], a subsequent study using an intact MHV genome demonstrated that the secondary structure is nonessential for RNA synthesis but plays a pivotal role in pathogenesis [21]. Downstream of these higher-order structures are 3′-terminal 55 nts and poly(A) tail. Although coronaviral poly(A) tail has been demonstrated to be a critical element for coronavirus replication [22], the role of the entire 3′-terminal 55 nts in the (+)-strand RNA synthesis remains to be determined.

In contrast, studies on the *cis*-acting RNA elements required for (−)-strand RNA synthesis in coronaviruses have been hampered by the limit amount of (−)-strand RNA present in the virus-infected cells and the lack of sufficient methods to detect (−)-strand coronaviral RNA in low numbers. For example, there are only an estimated ∼5 molecules of BCoV DI RNA (−) strand per cell in BCoV DI RNA-transfected BCoV-infected human rectal tumor (HRT)-18 cells at 24 hour postinfection (hpi) of virus passage 1 (VP1) [23], [24]. In order to overcome the detection deficiency and the false positive results of the (−)-strand DI RNA detection caused by artifact of (−)-strand DI RNA transcripts generated by T7 RNA polymerase using plasmid DNA as a template [25], a head-to-tail ligation method and reverse transcription polymerase chain reaction (RT-PCR) have been developed to detect (−)-strand BCoV DI RNA [26]. This method, therefore, may be employed to determine the requirements of *cis*-acting elements for the synthesis of BCoV (−)-strand DI RNA.

In this study we used head-to-tail ligation and real-time quantitative reverse transcription polymerase chain reaction (qRT-PCR) to detect and quantitate BCoV (−)-strand DI RNA synthesis. Using this technique along with Northern blot assay, we specifically defined the *cis*-acting elements within the 3′-terminal 55 nts that are required for the synthesis of (+)- or (−)-strand BCoV DI RNA by simultaneous comparison of their efficiency of RNA synthesis. Our findings suggest that the 3′-terminal 55 nts harbor *cis*-acting RNA elements required for both (+)- and (−)-strand RNA synthesis.

## Results

### The 3′-terminal 55 nts within the individual subgroups of betacoronaviruses were highly conserved

**Figure 1:**
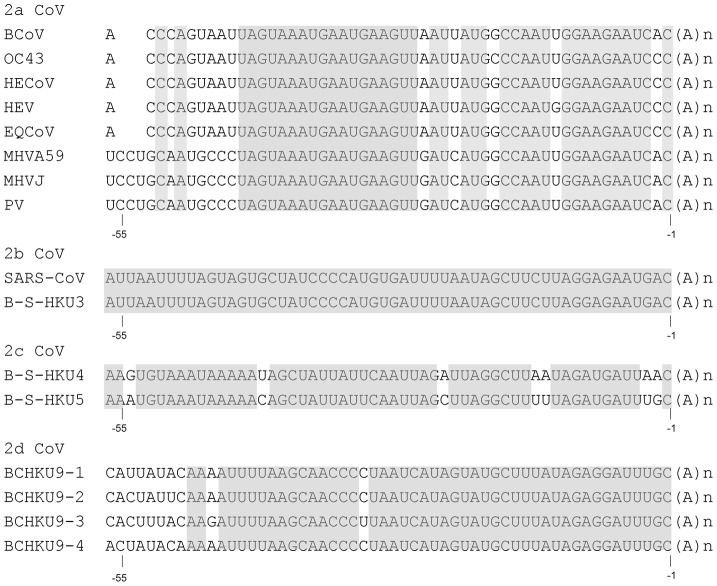
Alignment of the 3′-terminal 55-nt sequence within the individual subgroups of betacoronaviruses. The conserved sequence within individual subgroups is identified by shading. Position numbers −1 and −55 below the sequence indicate the first and 55th nt counted from the poly(A) tail, respectively. Abbreviations: CoV, coronavirus; BCoV, bovine coronavirus-Mebus; OC43, human coronavirus-OC43; HECoV-4408, human enteric coronavirus-4408; HEV, porcine hemagglutinating encephalomyelitis virus-TN11; ECoV, equine coronavirus-NC99; MHVA59, mouse hepatitis virus-A59; MHVJ, mouse hepatitis virus-JHM; PV, puffinosis virus; SARS-CoV, SARS coronavirus; B-S-HKU3, bat SARS coronavirus HKU3; B-S-HKU4, bat SARS coronavirus HKU4; B-S-HKU5, bat SARS coronavirus HKU5; BCHKU9-1, bat coronavirus HKU9-1; BCHKU9-2, bat coronavirus HKU9-2; BCHKU9-3, bat coronavirus HKU9-3; BCHKU9-4, bat coronavirus HKU9-4. GenBank Accession Nos. for the sequences studied here are as follows: U00735 for BCoV-Mebus, AF523847 for HCoV-OC43, AF523848 for HECoV-4408, AF523849 for HEV-TN11, AF523850 for ECoV-NC99, NC001846 for MHV-A59, X00990 for MHV-JHM, AJ544718 for PV, NC004718 for SARS-CoV, NC009694 for B-S-HKU3, NC009019 for B-S-HKU4, NC009020 for B-S-HKU5, EF065513.1 for BCHKU9-1, EF065514.1 for BCHKU9-2, EF065515.1 for BCHKU9-3, and EF065516.1 for BCHKU9-4.

Before the outbreak of severe acute respiratory syndrome (SARS), betacoronaviruses were considered to include one lineage. After the discovery of SARS-CoV, other novel subgroups of the betacoronaviruses have been proposed and the original group 2 coronaviruses, which included BCoV and MHV, became designated as 2a coronaviruses [27], [28]. Many *c*is-acting elements in the 3′ UTR of BCoV and MHV-A59 have been characterized [12], [13], [15], [22], [29]. To examine whether the 3′-most 55 nts have conserved features within the individual subgroups of betacoronaviruses, the 3′-terminal 55-nt sequence was aligned according to the published sequences (see GenBank Accession Nos. in Fig. 1) and the sequence identity within the subgroup 2a, 2b, 2c and 2d coronaviruses was 71%, 100%, 88% and 82%, respectively (Fig. 1). It is apparent that the aligned 3′-terminal 55-nt sequence was highly conserved (identified by shading) across the coronaviruses within the individual subgroups of betacoronaviruses, suggesting that this region may harbor *cis*-acting elements critical for coronavirus replication.

### The 3′-terminal 55 nts on BCoV DI RNA are required for (−)-strand RNA synthesis as analyzed by head-to-tail RNA ligation and qRT-PCR

**Figure 2:**
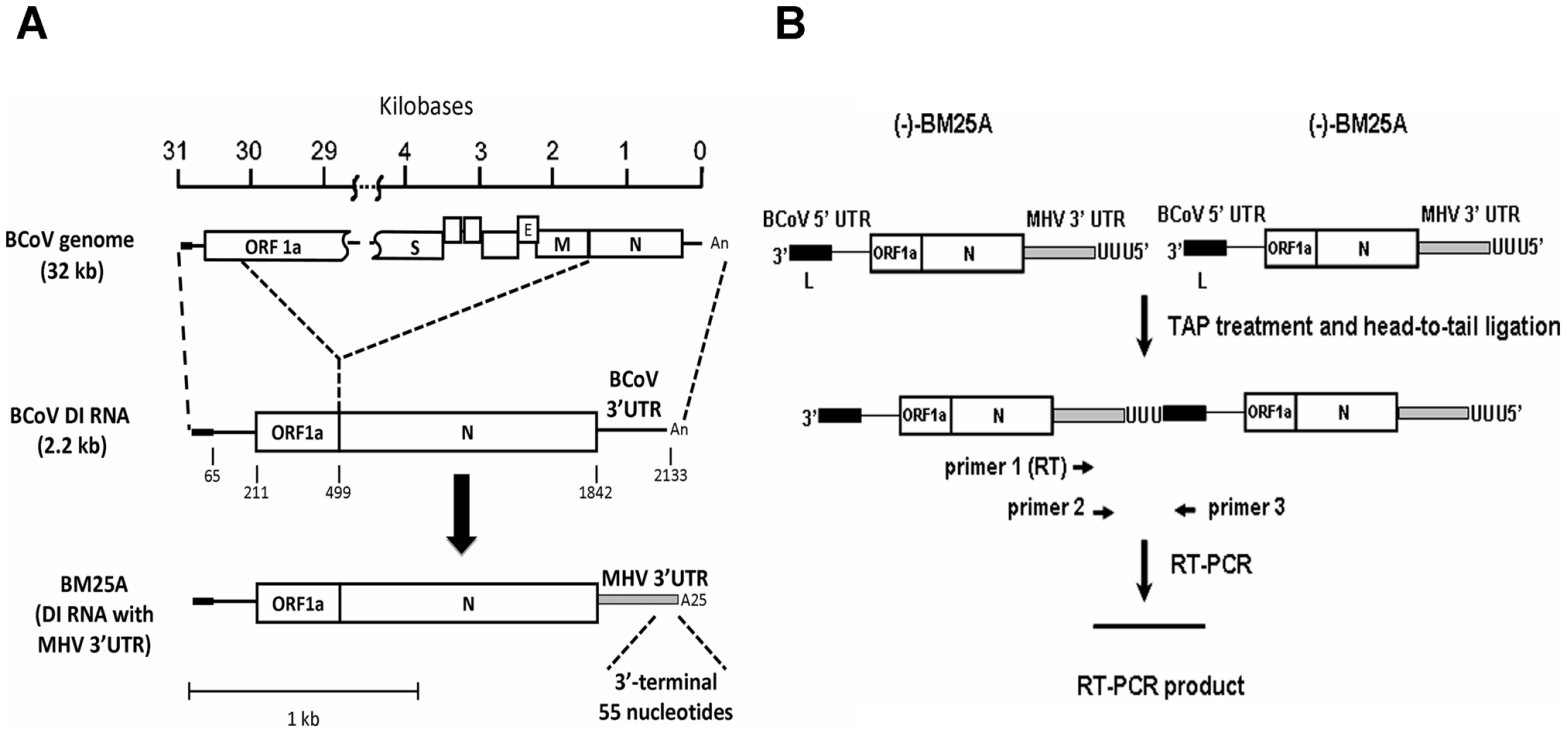
Diagram of the hybrid BCoV DI RNA and the head-to-tail strategy used to detect the synthesis of (−)-strand DI RNA. (A) Upper panel: the composition of naturally occurring BCoV DI RNA relative to the full-length BCoV genome. Lower panel: the modified BCoV DI RNA construct BM 25A used in this study. The 65-nt leader sequence is illustrated with a filled rectangle. (B) Strategy for determining the synthesis of (–)-strand DI RNA. RNA extracted at 8 hpt of DI RNA transcript was treated with TAP and head-to-tail ligated. MHV-A59 3′ UTR-negative-strand-specific primer 1 MHV3UTR3(–) was used for RT with the ligated RNA as template. MHV-A59 3′ UTR-negative-strand-specific primer 2 MHV3UTR6(–) and BCoV 5′ UTR-positive-strand specific primer 3 BCoV23-40(+) were used for PCR.

**Figure 3:**
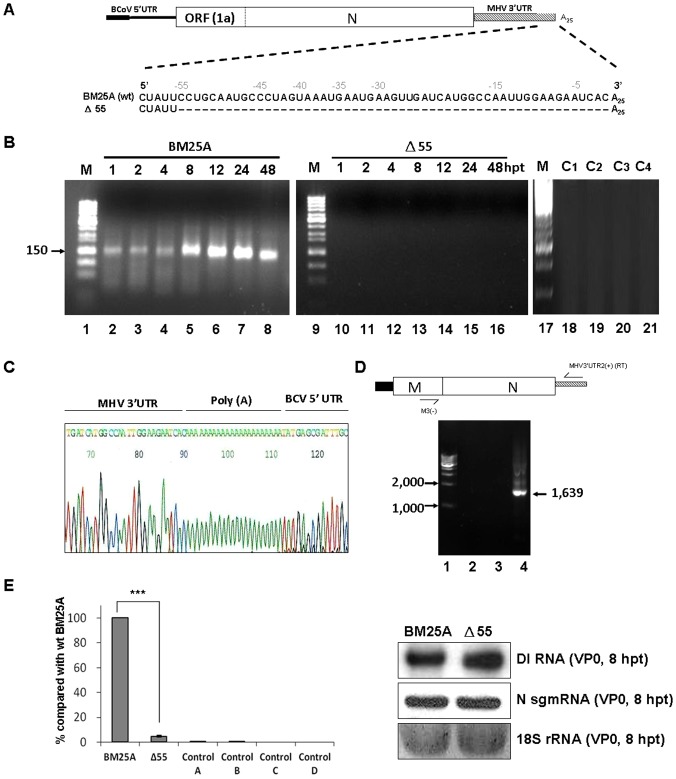
Analysis of the requirement of 3′-terminal 55 nts for the synthesis of (−)-strand BCoV DI RNA. (A) Diagram of the BCoV DI RNA BM25A with the intact 3′-terminal 55 nts and the mutant construct Δ55 with the deletion of 3′-terminal 55 nts (denotes with dashes). (B) Detection of (–)-strand BCoV DI RNA with head-to-tail ligation and RT-PCR. RT-PCR products with a size of ∼150 bp were observed from BCoV-infected BM25A-transfected cells (lanes 2–8, arrowhead) but not from BCoV-infected Δ55-transfected cells (lanes 10-16). Lanes 18–21 represent the controls for RT-PCR. C1: total cellular RNA from mock-infected cells. C2: total cellular RNA from BCoV-infected cells. C3: total cellular RNA from DI RNA-transfected mock-infected cells. C4: a mixture of BCoV-infected cellular RNA extracted at 10 hpi and 200 ng of BM25A transcript. (C) Sequence of the cDNA-cloned RT-PCR product with a size of ∼150 bp from lane 5 in Fig. 3B. [shown in the (+)-strand]. (D) Detection of the potential recombination between the helper virus genome and DI RNA. The primers MHV3′ UTR2(+), which anneals to the MHV 3′ UTR and was used for RT, and M3(–), which anneals to BCoV M protein gene, were used for PCR to detect potential recombination between helper virus BCoV genome and BM25A (lane 2) or Δ55 (lane 3). A recombinant DNA of 1,639 nt shown in lane 4 was created by overlap RT-PCR and was used as a size marker for the product generated with the primers MHV 3′ UTR2(+) and M3(–). (E) Left panel: the relative levels of (–)-strand DI RNA synthesis as measured by qRT-PCR. Control A: total cellular RNA from mock-infected cells. Control B: total cellular RNA from BCoV-infected cells. Control C: total cellular RNA from DI RNA-transfected mock-infected cells. Control D: a mixture of BCoV-infected cellular RNA extracted at 10 hpi and 200 ng of BM25A transcript. Right panel: the amounts of DI RNA, helper virus N sgmRNA, and 18S rRNA from DI RNA-transfected BCoV-infected cells at 8 hpt of VP0 as measured by Northern blot assay. The values (E) represent the mean ±SD of three individual experiments. SD: standard deviation. ***p<0.001.

The coronavirus DI RNA has been employed as a surrogate for coronavirus genome to study the *cis*-acting elements required for coronavirus replication [3], [5], [6], [11], [15], [20], [22], [29], [30]. The identification of *cis*-acting RNA elements that are essential for the (−)-strand DI RNA synthesis in coronaviruses, however, has been restricted by the low numbers of (−)-strand DI RNA produced from the DI RNA-transfected helper virus-infected cells [23], [24]. Although RT-PCR may be used to detect the (−)-strand DI RNA molecules, a false positive result may occur due to the synthesis of the copy-back (−)-strand DI RNA transcripts generated by T7 RNA polymerase *in vitro* using plasmid DNA as a template [25]. Thus, to overcome the detection problems caused by the low numbers of (−)-strand DI RNA and to avoid the detection of the (−)-strand DI RNA resulting from the copy-back transcripts, we, based on the previous study [26], (1) created a hybrid BCoV DI RNA construct marked with the MHV 3′ UTR and 25 nts of poly(A) tail (Fig. 2A) to distinguish it from BCoV helper virus genome during RT-PCR and (2) conducted head-to-tail ligation of RNA and RT-PCR to differentiate the coronaviral polymerase-generated (−)-strand DI RNA from T7 RNA polymerase-generated artifact (−)-strand DI RNA (Fig. 2B), as the primers used in the RT-PCR cannot yield a product from the copy-back (−)-strand transcripts after ligation [26]. Note that the sequence in the 3′-termianl 55 nts of the 3′UTR between BCoV and MHV is highly conserved (Fig. 1) and it has been shown that the replication efficiency of BCoV DI RNA with the MHV 3′ UTR is almost the same with that of BCoV DI RNA with BCoV 3′UTR [26]. Accordingly, to test whether the method can be applied to examine the requirement of the 3′-most 55 nts for (−)-strand DI RNA synthesis in the context of BCoV DI RNA, BM25A construct in which the 3′-most 55 nts were intact and Δ55 mutant in which the 3′-most 55 nts were deleted were created (Fig. 3A). After transfection of BM25A and Δ55 transcripts into BCoV-infected HRT-18 cells, RNA was extracted at different time points as indicated in Fig. 3B, head-to-tail ligated, and subjected to RT-PCR. An RT-PCR product with a length of ∼150 base pair (bp) was obtained from BM25A-transfected BCoV-infected cells (Fig. 3B, lanes 2–8) but not from Δ55-transfected BCoV-infected cells (Fig. 3B, lanes 10–16), mock-infected cells (Fig. 3B, lane 18), BCoV-infected cells (Fig. 3B, lane 19), BM25A-transfected mock-infected cells (Fig. 3B, lane 20), or a mixture of BCoV-infected cellular RNA extracted at 10 hpi and 200 ng of BM25A transcript (Fig. 3B, lane 21). These results, along with sequencing analysis (Fig. 3C), confirmed that the ∼150-bp RT-PCR product obtained from BM25A-transfected BCoV-infected cells was specific from head-to-tail-ligated coronavirus polymerase-generated (–)-strand DI RNA. Because DI RNA may undergo recombination with helper virus genome under select pressure [31], [32], [33], [34], the detected (–)-strand DI RNA in Fig. 3B may be derived from the recombinant genome rather than input (+)-strand DI RNA. To seek evidence of a DI RNA reporter-containing recombinant genome, the RNA samples extracted from the BM25A- and Δ55-transfected BCoV-infected cells at 8 hour posttransfection (hpt) were tested for a 1,639-nt helper virus genome-integrated reporter-containing sequence between the membrane (M) protein gene and the DI RNA 3′ UTR using RT-PCR [26]. No RT-PCR product (Fig. 3D, lanes 2–3) was observed, suggesting that the detected ∼150 bp RT-PCR products do not result from a recombinant helper virus genome. To compare the efficiency of (–)-strand DI RNA synthesis between BM25A and Δ55, head-to-tail ligated RNA extracted at 8 hpt was subjected to qRT-PCR with the same primer set used for the RT-PCR analysis. The results (Fig. 3E, left panel) suggest that the efficiency of (–)-strand DI RNA synthesis from Δ55 was significantly impaired in comparison with that from BM25A. Thus, we conclude that (1) using the head-to-tail RNA ligation and qRT-PCR allows us to detect and quantitate (–)-strand BCoV DI RNA and (2) the 3′-most 55 nts in BCoV DI RNA are required for (–)-strand DI RNA synthesis.

### Identification of *cis*-acting RNA elements within the 3′-terminal 55 nts for (−)- and (+)-strand DI RNA synthesis

**Figure 4:**
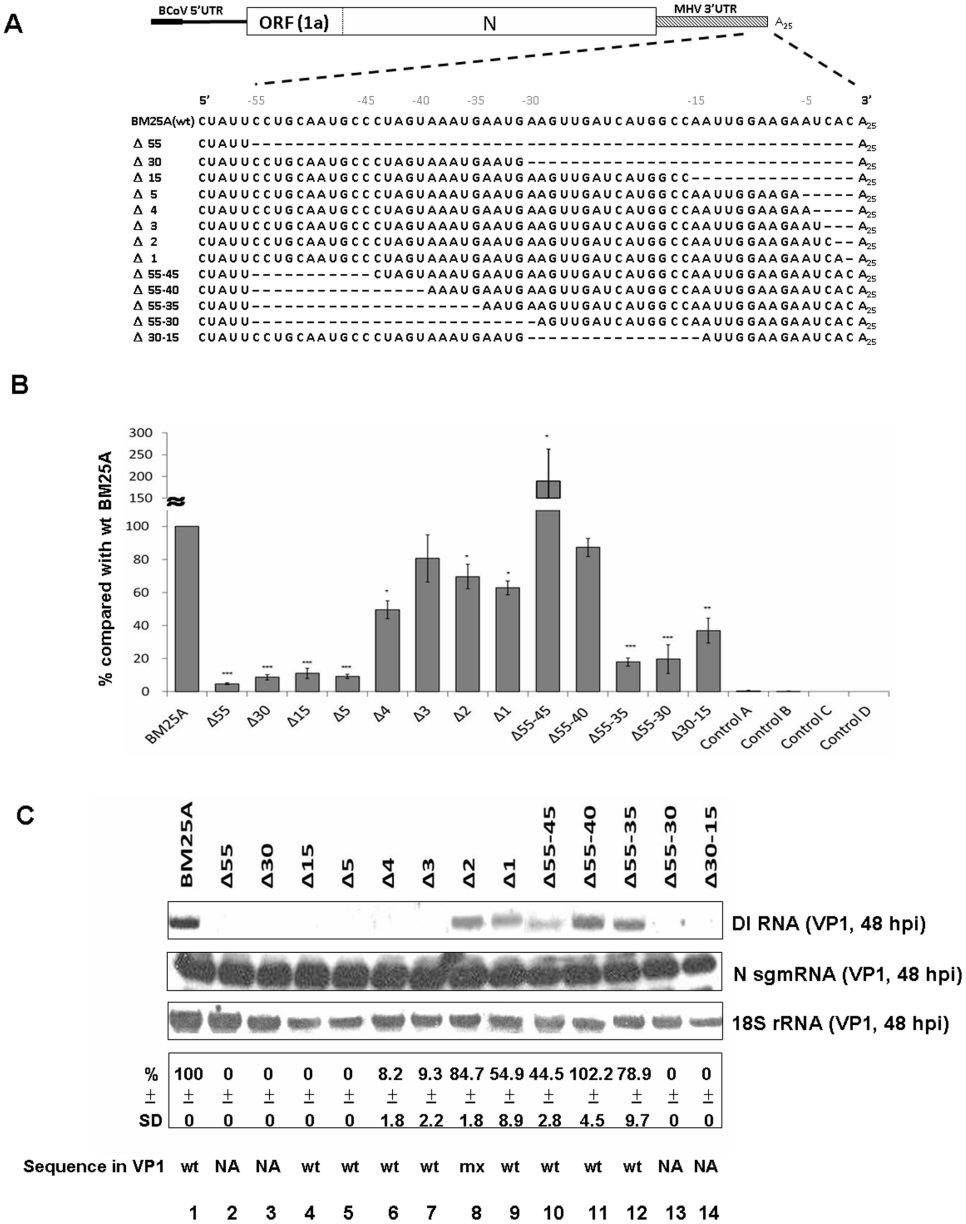
Identification of *cis*-acting RNA elements within the 3′-terminal 55 nts that are required for (−)- and (+)-strand RNA synthesis. (A) Constructs of deletion mutants within the 3′-terminal 55 nucleotides of BCoV DI RNA. Dashes denote deleted sequences. (B) The relative levels of (–)-strand DI RNA synthesis. Total cellular RNA was extracted from DI RNA-transfected BCoV-infected cells at 8 hpt. The synthesis of (–)-strand DI RNA from the deletion mutant was quantitated by qRT-PCR and was compared with that from wt BM25. Control A: total cellular RNA from mock-infected cells. Control B: total cellular RNA from BCoV-infected cells. Control C: total cellular RNA from DI RNA-transfected mock-infected cells. Control D: a mixture of BCoV-infected cellular RNA extracted at 10 hpi and 200 ng of BM25A transcript. (C) Upper panel: the synthesis of (+)-strand DI RNA as detected by Northern blot assay. Total cellular RNA was extracted at 48 hpi of VP1 and was subjected to Northern blot assay with N sgmRNA and 18S rRNA as internal controls. Middle panel: the relative levels of the (+)-strand DI RNA synthesis. Lower panel: the sequence of the BCoV DI RNA at 48 hpi of VP1 as determined by RT-PCR and sequencing analysis. The values (B) and (C) represent the mean ±SD of three individual experiments. SD: standard deviation, wt: wild type, mx: mixed, NA: not available. *p<0.05, **p<0.01, ***p<0.001.

We have demonstrated in the present study that the 3′-most 55 nts are required for the synthesis of (–)-strand BCoV DI RNA using the head-to-tail RNA ligation and qRT-PCR (Fig. 3). The map positions of the *cis*-acting RNA elements required for (–)- and (+)-strand DI RNA synthesis within this 55-nt region, however, remain to be determined. To define the specific *cis*-acting RNA elements within the 3′-terminal 55 nts which function in the synthesis of BCoV DI RNA (–) or (+) strands, a series of DI RNA constructs with deletions either from the 3′ or 5′ end of the 55-nt region were generated by mutagenesis (Fig. 4A) and tested by the methods depicted in Fig. S1 and described as follows. The T7 RNA polymerase-generated transcript of each construct was transfected into BCoV-infected HRT-18 cells and virus within the transfected cells was referred to as virus passage 0 (VP0). The total cellular RNA was collected at 8 hpt of RNA transcript to detect the synthesis of (–)-strand DI RNA by qRT-PCR. Since the transfected transcript was still in the BCoV-infected cells at 8 hpt as shown in Fig. 3E (right panel), it is difficult to differentiate between the coronaviral polymerase-generated (+)-strand DI RNA and T7 RNA polymerase-generated (+)-strand DI RNA by Northern blot assay at this time point. Therefore, to determine whether the detected (+)-strand DI RNA is specifically synthesized by the BCoV polymerase, the supernatant virus collected at 48 hpt, which is now called virus passage 1 or VP1, was used to infect fresh HRT-18 cells (Fig. S1). The total cellular RNA collected at 48 hpi of VP1 was used to detect the synthesis of (+)-strand DI RNA by the Northern blot assay. If the T7 RNA polymerase-generated DI RNA, which is transfected at VP0, is not able to replicate, the transfected (+)-strand DI RNA cannot be detected at 48 hpi of VP1 [5], [6], [15], [35], [36]. The signals detected by Northern blot assay were quantitated and presented in percentages as a result of (+)-strand DI RNA synthesis [relative to the wt BM25A (100%)]. These results are summarized in Figs 4B and 4C. As shown in Fig. 4B, DI RNA constructs with a 1-, 2-, 3-, and 4-nt deletion (constructs Δ1, Δ2, Δ3 and Δ4, respectively) at the 3′ terminus of the 55-nt region still maintained the synthesis of (–)-strand DI RNA (Fig. 4B) although the synthesis efficiency was impaired (∼55% to ∼80% of the wt BM25A). Among these constructs, BCoV DI RNA with a 1- and 2-nt deletion at the 3′ terminus still supported the (+)-strand synthesis (54.9% and 84.7% of the wt BM25A, respectively) (Fig. 4C, lanes 8–9), whereas DI RNA constructs with a 3- and 4-nt deletion totally abolished the (+)-strand DI RNA synthesis (Fig. 4C, lanes 6–7). Further deletion of 5-, 15-, 30-, and 55- nts (constructs Δ5, Δ15, Δ30 and Δ55, respectively) (Fig. 4A) from the 3′ terminus of the 55-nt region led to a significant decrease in the synthesis of (–)-strand (less than 10% of wt BM25A) (Fig. 4B) and (+)-strand DI RNA (Fig. 4C, lanes 2–5).

**Figure 5:**
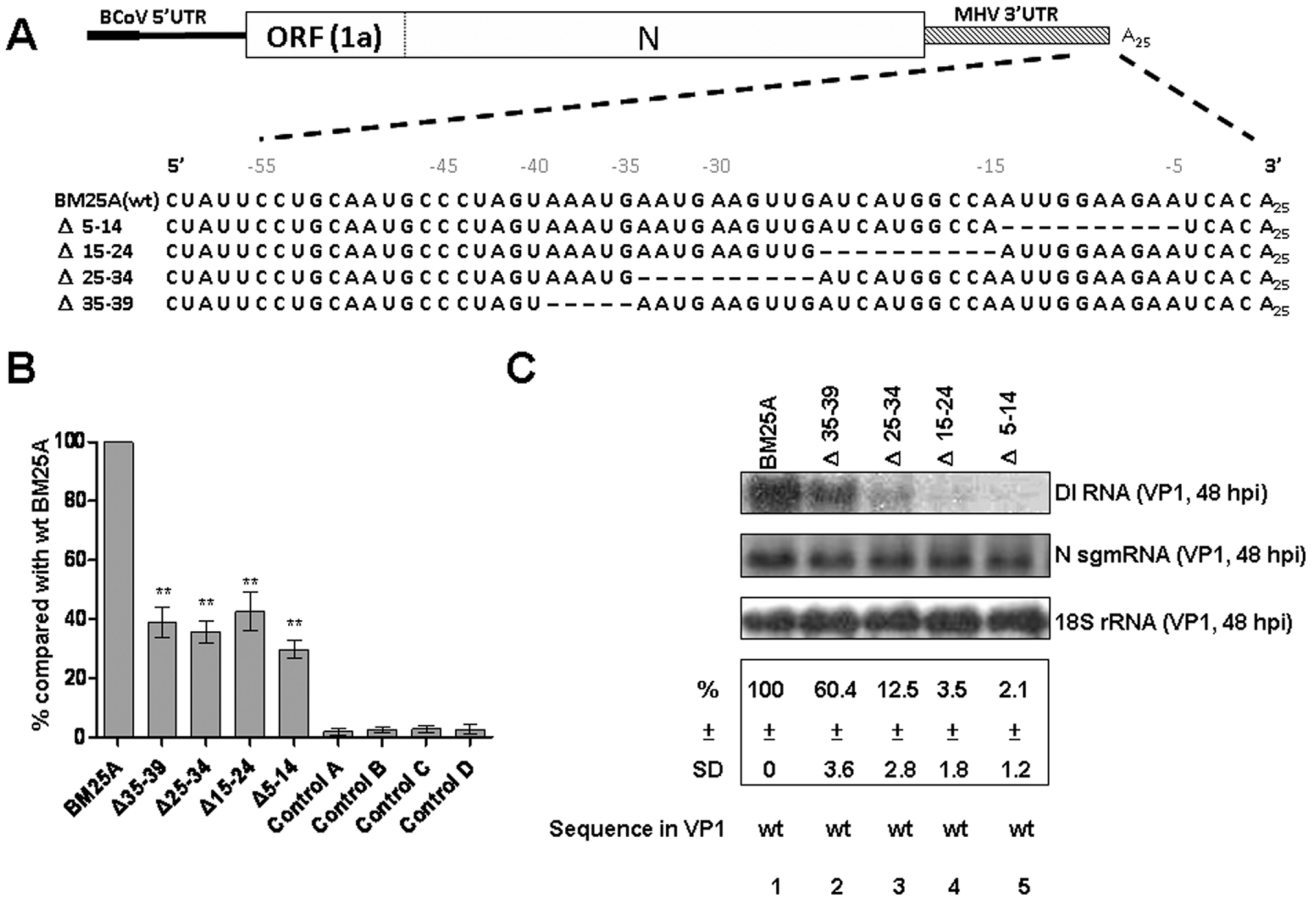
Determination of *cis*-acting RNA elements between the nts −4 and −40 required for (−)- and (+)-strand RNA synthesis. (A) Deletion mutants of BCoV DI RNA. Dashes denote deleted sequences. (B) The relative levels of (−)-strand DI RNA synthesis. The synthesis of (−)-strand DI RNA from the deletion mutant was quantitated by qRT-PCR and was compared with that from wt BM25. Control A: total cellular RNA from mock-infected cells. Control B: total cellular RNA from BCoV-infected cells. Control C: total cellular RNA from DI RNA-transfected mock-infected cells. Control D: a mixture of BCoV-infected cellular RNA extracted at 10 hpi and 200 ng of BM25A transcript. (C) Upper panel: the synthesis of (+)-strand DI RNA as detected by Northern blot assay. Total cellular RNA was extracted at 48 hpi of VP1 and was subjected to Northern blot assay with N sgmRNA and 18S rRNA as internal controls. Middle panel: the relative levels of the (+)-strand DI RNA synthesis. Lower panel: the sequence of the BCoV DI RNA at 48 hpi of VP1 as determined by RT-PCR and sequencing analysis. The values (B) and (C) represent the mean ±SD of three individual experiments. SD: standard deviation, wt: wild type. **p<0.01.

To further dissect the specific *cis*-acting RNA motif required for (–)- and (+)-strand RNA synthesis within this 55-nt region, DI RNA constructs with various deletions from the 5′ terminus of this region were created and tested (Fig. 4A). The efficiency of (–)-strand DI RNA synthesis from DI RNA with a deletion from nts –55 to –45 (construct Δ55-45) was not affected and was even better than that from wt BM25A (Fig. 4B) although the efficiency of (+)-strand DI RNA synthesis was impaired (44.5% of wt BM25A, Fig. 4C, lane 10). The extension of the deletion from nts -55 to -40 in DI RNA (construct Δ55-40) still did not affect the efficiency of both (–)-and (+)-strand DI RNA synthesis (Fig. 4 B and Fig. 4C, lane 11, respectively). Surprisingly, DI RNA with a deletion from nts –55 to –35 (construct Δ55-35) almost blocked the synthesis of (–)-strand DI RNA (less than 20% of wt BM25A, Fig. 4B) but still maintained the (+)-strand DI RNA synthesis (78.9% of wt BM25A, Fig. 4C, lane 12). On the other hand, both (–)- and (+)-strand DI RNA synthesis were significantly inhibited from DI RNA with a deletion from nts –55 to –30 (construct Δ55-30) (Fig. 4B and Fig. 4C, lane 13, respectively). The efficiency of (–)-strand DI RNA synthesis was also significantly blocked in the construct Δ30-15 with a deletion from nts –30 to –15 (∼40% of wt BM25A, Fig. 4B) and no (+)-strand DI RNA synthesis was detected from this deletion mutant (Fig. 4C, lane 14), suggesting the nts from –30 to –15 are critical for both (–)- and (+)-strand DI RNA synthesis. Of these deletion mutants, all of the mutated sequences in the synthesized (–)-strand DI RNA were still maintained at 8 hpt; however, the available mutant sequences in the (–)- and (+)- strand DI RNA at 48 hpi of VP1 showed that all contained the sequence with majority from wt DI RNA except for the mutant Δ2 in which the sequence of mutant Δ2 was mixed with that of wt BM25A (Fig. 4C). Based on these results, the tentative conclusions at this time were as follows: (1) the nts from –1 to –4 and from –40 to –55 are not important for the (–)-strand BCoV DI RNA synthesis and (2) the nts from –1 to –2 and from –35 to –55 are not absolutely required for the (+)-strand BCoV DI RNA synthesis. Therefore, the critical *cis*-acting elements essential for (–)- or (+)-strand synthesis may reside in the region between the nts –2 and –40. To further determine the requirement of *cis*-acting elements for the (–)- and (+)-strand BCoV DI RNA synthesis in this region, deletion mutants Δ5–14, Δ15–24, Δ25–34, and Δ35–39 were constructed (Fig. 5A) and tested. As shown in Fig 5B, the efficiency of the (−)-strand DI RNA synthesis from these deletion constructs was inhibited (∼29% to ∼42% of wt BM25A). The (+)-strand DI RNA synthesis was still maintained from construct Δ35–39 (60.4% of wt BM 25A) (Fig. 5C, lane 2) but was significantly blocked from constructs Δ5–14, Δ15–24 and Δ25–34 (Fig. 5C, lanes 3–5). Sequencing analysis showed that all of the mutated sequences in these synthesized (−)-strand DI RNA were still maintained at 8 hpt; however, all the mutants reversed back to wt DI RNA at 48 hpi of VP1. The sequencing results acquired from DI RNA constructs Δ1, Δ55–45, Δ55–40, Δ55–35, and Δ35–39 at 48 hpi of VP1 (Figs. 4C and 5C) indicate that the (+)-strand DI RNA detected by Northern blot assay may mostly originated from wt DI RNA (BM25A) that likely arose by reversion and consequently the importance of the mutated sequences remains to be determined. To confirm that these DI RNA mutants are able to replicate, the supernatant collected at 48 hpt was used to infect fresh HRT-18 cells. The total cellular RNA was harvested at 24 hpi of VP1 and then subjected to Northern blot assay and sequencing analyses. As shown in Fig. S2, the relative replication efficiency of these mutants at 24 hpi of VP1 was similar to that at 48 hpi of VP1. Sequencing analysis revealed that the mutated sequences were still maintained in the mutants Δ2, Δ55–40 and Δ35–39. Although the sequencing results presented a mixed population in the mutants Δ1, Δ55–45 and Δ55–35, the majority of the sequences were from the mutants rather than from the wt DI RNA (BM25A). These results suggest that these DI RNA mutants still maintain the ability to replicate although the replication efficiency among these mutants varied. Taken together, based on the deletion mutagenesis experiments and sequence analysis, we conclude that the nts from −5 to −39 within the 3′-terminal 55 nts are the *cis*-acting elements responsible for the (−)-strand BCoV DI RNA synthesis and that the nts from −3 to −34 within the 3′-terminal 55 nts are the *cis*-acting elements required for the (+)-strand BCoV DI RNA synthesis.

### The effect of the 3′-most nucleotide species on the (−)- and (+)-strand DI RNA synthesis

**Figure 6:**
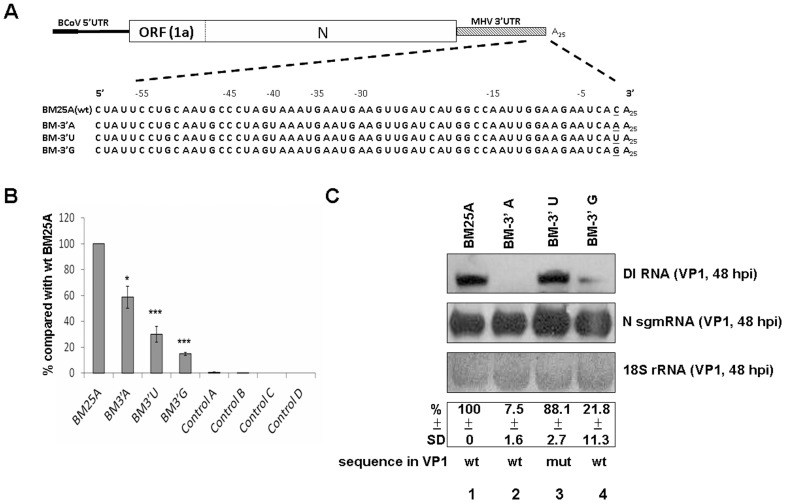
Effect of nucleotide species at the -1 position of 3′ terminal sequence in BCoV DI RNA on (−)- and (+)-strand RNA synthesis. (A) DI RNA constructs with nucleotide substitution (underlined) at the −1 position of 3′ terminal sequence. (B) The relative levels of (−)-strand DI RNA synthesis. BCoV-infected HRT-18 cells were transfected with the indicated DI RNA construct at 2 hpi, and the total cellular RNA was extracted at 8 hpt. The synthesis of the (−)-strand DI RNA from the substitution mutant was quantitated by qRT-PCR and compared with that from wt BM25A. Control A: total cellular RNA from mock-infected cells. Control B: total cellular RNA from BCoV-infected cells. Control C: total cellular RNA from DI RNA-transfected mock-infected cells. Control D: a mixture of BCoV-infected cellular RNA extracted at 10 hpi and 200 ng of BM25A transcript. (C) Upper panel: the synthesis of (+)-strand DI RNA as detected by Northern blot assay with N sgmRNA and 18S rRNA as internal controls. Middle panel: the relative levels of (+)-strand DI RNA synthesis. Lower panel: the sequence of the BCoV DI RNA at 48 hpi of VP1 as determined by RT-PCR and sequencing analysis. The values (B) and (C) represent the mean ±SD of three individual experiments. SD: standard deviation, wt: wild type, mut: mutant. *p<0.05, ***p<0.001.

Little is known regarding the mechanism of the initiation of (−)-strand RNA synthesis in coronaviruses. Since the last nucleotide of the 3′ UTR in all coronavirus genomes sequenced to date is cytosine, it was speculated that this conserved nucleotide species may play an important role in coronavirus replication. To test this hypothesis, the cytosine residing at the last nucleotide of the 3′ UTR in BCoV DI RNA was substituted with adenine, uracil and guanine to make the constructs BM-3′A, BM-3′U, and BM-3′G, respectively (Fig. 6A). As shown in Fig. 6B, even though construct BM-3′A was still able to synthesize its (−)-strand counterpart (∼60% of wt BM25A), its ability to synthesize (+)-strand was significantly abolished (7.5% of wt BM25A) (Fig. 6C, lane 2). In contrast, the efficiency of the (−)-strand DI RNA synthesis from construct BM-3′U was significantly inhibited (∼30% of wt BM25A) (Fig. 6B), but the efficiency of (+)-strand DI RNA synthesis from construct BM-3′U was similar to that from wt BM25A (88.1% of wt BM25A, Fig. 6C, lane 3). The efficiency of both (−)- and (+)-strand DI RNA synthesis (Fig. 6B and Fig. 6C, lane 4, respectively) from construct BM-3′G, on the other hand, was significantly blocked (∼10% and ∼21.8% of wt BM25A, respectively). Sequencing analysis from VP1 revealed that the mutants BM-3′A and BM-3′G reverted back to the wt sequence, but the mutant BM-3′U still maintained the mutated sequence. The results together suggest that (1) the substitution of the 3′-most nucleotide cytosine with other nucleotide species is sensitive for (−)-strand DI RNA synthesis and (2) the substitution of the 3′-most cytosine residue by uracil, but not adenine or guanine, is more tolerant for (+)-strand DI RNA synthesis. The nucleotide species at the 3′-most position (−1), therefore, is important, but not critical, for both (−)- and (+)-strand BCoV DI RNA synthesis.

## Discussion

Many *cis*-acting elements required for coronavirus replication have been identified in the past two decades [3], [4], [5], [6], [7], [8], [9], [10], [11], [12], [13], [14], [15], [16], [17]. The identification of the *cis*-acting elements specific for (−)-strand RNA synthesis in coronavirus, however, has been hampered due to insufficiencies in the techniques used to detect and quantitate RNA (−) strand species. In this study, we employed the method of head-to-tail ligation and qRT-PCR to examine the *cis*-acting elements required for (−)-strand BCoV DI RNA synthesis [26], [37]. This method has the advantages of detecting and quantitating low copy number of (−)-strand BCoV DI RNA as well as distinguishing between T7 RNA polymerase-generated (−)-strand DI RNA transcripts and coronaviral polymerase-generated (−)-strand DI RNA, and thereby allows us to systematically examine the *cis*-acting RNA elements required for (−)-strand DI RNA synthesis and to gain insight into the mechanism of coronavirus replication.

Replication of (+)-strand RNA viruses can be generally divided into two steps. First, the viral polymerase employs a (+)-strand genome as a template to generate the (−)-strand complement. Second, the synthesized (−)-strand genome then serves as a template for the synthesis of the (+)-strand counterpart. The majority of previous studies on *cis*-acting elements in coronaviruses emphasize their roles in replication [interpreted as (+)-strand synthesis]; however, it is still unclear that the identified *cis*-acting elements function in (−)- or (+)-strand synthesis. We in this study, therefore, simultaneously analyzed the effects of *cis*-acting elements on the synthesis of both (−)- and (+)-strand BCoV DI RNA during infection in order to specifically define the role of a *cis*-acting element. According to the results concerning the (−)- and (+)- strand DI RNA synthesis from mutants Δ5, Δ15, Δ30 and Δ55, we conclude that the deleted sequences may function at least in the (−)-strand DI RNA synthesis because the deleted sequences significantly blocked the (−)-strand DI RNA synthesis from their (+)-strand DI RNA templates (Fig. 4B). Regarding the role of these deleted sequences in the (+)-strand DI RNA synthesis, however, it is not feasible to speculate from these mutants at this time because the undetectable (+)-strand DI RNA in VP1from these mutants may be due to the very low amounts of the (−)-strand DI RNA templates or due to the deleted sequences which block the synthesis of (+)-strand DI RNA. For the mutants Δ1, Δ2, Δ3, and Δ4, the (−)-strand DI RNA synthesis was overall slightly affected with the deleted sequences (Fig. 4B). However, the defect in (+)-strand DI RNA synthesis from the mutants Δ3 and Δ4 (Fig. 4C, lanes 6–7) indicates that nts from −3 to −4 are critical *cis*-acting elements in the synthesis of (+)-strand rather than (−)-strand DI RNA. In comparison with wt BM25A, the efficiency of both (−)- and (+)-strand DI RNA synthesis from the mutants Δ55–45 and Δ55–40 was not significantly inhibited, suggesting nts from −55 to −40 are not absolutely required for both (−)- and (+)-strand DI RNA synthesis. Interestingly, the efficiency of (−)-strand RNA synthesis from deletion mutant Δ55-45 was even higher than that from wt BM25A as shown in Fig. 4B. Regarding the 3′-terminal 55 nts in the MHV 3′ UTR, the sequence between −1 and −44 in the deletion mutant Δ55-45 is nearly identical to that in the BCoV 3′ UTR after alignment. It could be possible that BCoV RNA-dependent RNA polymerase utilizes the almost authentic BCoV 3′ UTR in mutant Δ55-45 more efficiently than the MHV 3′ UTR in the BM25A, leading to a better efficiency in the synthesis of (−)-strand DI RNA. It is also intriguing that the replication efficiency from the 16-nts deletion mutant Δ55-40 was enhanced, but the replication efficiency from the 11-nts deletion mutant Δ55-45 was repressed in comparison with that from wt BM25A (Fig. 4C). However, the involvement of 3′ UTR elements such as enhancers and repressors in the regulatory functions of replication has been suggested in RNA viruses [38], [39], [40]. Therefore, considering the replication efficiency of Δ55–45, Δ55–40 and Δ55–35 (44%, 102% and 78%, respectively), we speculate that nts −45 to −55 may serve as an enhancer because the deletion of these nts in mutant Δ55–45 leads to the modest inhibition of replication. In contrast, nts −40 to −44 may act as a repressor and the synergistic effect of both enhancer and repressor, which are missing in the mutant Δ55–40, could be the reason why the construct had the similar or even higher replication efficiency in comparison with wt BM25A. Similarly, nts −35 to −39 may play a role of enhancer as evidenced by the comparison of replication efficiency between Δ55–40 and Δ55–35 (102% vs 78%) (Fig. 4C) and between wt BM25A and Δ35–39 (100% vs 60%) (Fig. 5C). In mutants Δ55–35 and Δ35–39, the efficiency of (−)-strand DI RNA synthesis was significantly decreased, but the (+)-strand DI RNA synthesis was still maintained. The results suggest that the efficiency of (+)-strand DI RNA synthesis may not be affected by the existing numbers of (−)-strand DI RNAs as long as the *cis*-acting element [on (−)-strand DI RNA] required for (+)-strand DI RNA synthesis is intact. These results are also in agreement with the previously proposed model which indicated that the (−)-strand viral RNA is repeatedly used as a template for (+)-strand viral RNA synthesis [29]. Based on the criteria, the deleted sequences in mutants Δ5–14, Δ15–24 and Δ25–34 are required for both (−)- and (+)-strand DI RNA synthesis since the (−)- and (+)-strand DI RNA synthesis from these mutants was significantly inhibited (Figs. 5B and 5C). Therefore, simultaneously analyzing the efficiency of (−)- and (+)-strand DI RNA synthesis enables us to specifically define the *cis*-acting elements that function in (−)- or (+)-strand DI RNA synthesis.

Because the last nucleotide of the 3′ UTR in all coronavirus genomes sequenced to date is cytosine and coronavirus nonstructural protein 8 has been proposed to preferentially initiate the synthesis of primer at the cytosine within the internal 5′-(G/U)CC-3′ trinucleotides [41], we in the present study speculated that the cytosine residue may play a critical role in (−)-strand RNA synthesis. Since the substitution of the cytosine with adenine, guanine or uracil inhibited the (−)-strand DI RNA synthesis at different levels (Fig. 6B), the 3′-most nucleotide species apparently is critical for (−)-strand DI RNA synthesis. Nevertheless, whether the cytosine is involved in the primer-dependent (−)-strand RNA synthesis remains to be elucidated. On the other hand, the results that the substitution of the cytosine with adenine and guanine, but not uracil, almost blocked (+)-strand DI RNA synthesis suggest that a purine (adenine and guanine), but not a pyrimidine (cytosine and uracil), on the 5′-most nucleotide of (−)-strand 3′ UTR is preferred for subsequent (+)-strand synthesis. Thus, these results suggest that the 3′-most nucleotide species on the DI RNA (+) strand is an important element for both (−)-and (+)-strand DI RNA synthesis.

The initiation of (−)-strand RNA synthesis in (+)-strand RNA viruses occurs at the 3′ terminal sequences of (+)-strand RNA, and consequently alterations of the 3′-terminal sequences of (+)-strand RNA may be detrimental to (−)-strand RNA synthesis as evidenced by this study and others [42], [43], [44], [45], [46], [47], [48]. However, evidence from this study and previous findings [12], [13], [14], [15] has also shown that alterations of the sequence at the 3′ terminal region of (+)-strand RNA [5′ terminal region on (−)-strand RNA] also impairs the synthesis of (+)-strand RNA. The reasons why the sequence alterations at the 3′-terminal region on (+)-strand RNA, for example, mutants Δ3, Δ4, BM-3′A, and BM-3′G, affected the synthesis of (+)-strand RNA remains unknown. However, since (1) the interactions between the 5′ and 3′ end of the genome facilitated by RNA or/and protein to form the replication complex and thereby to initiate replication have been well documented in numerous of RNA viruses [49], [50], [51], [52], [53], [54] and (2) the results from this study that the mutations in the 3′-terminal region of (+)-strand DI RNA were able to alter the efficiency of (+)-strand DI RNA synthesis, we postulate that the sequence mutations in the 3′-terminal region of (+)-strand DI RNA [5′ terminal region of (−)-strand DI RNA] may hinder the binding of proteins to this region and then block the interactions between the 5′ and 3′ end of the (−)-strand genome to constitute the replication complex that is required for (+)-strand synthesis. This notion is supported by the previous studies in which the nts from −26 to −36 in the MHV 3′ UTR have been demonstrated to be required for host protein binding and viral RNA replication [18], [55]. Further study is required to elucidate whether the *cis*-acting elements identified in this study and others are involved in the constitution of replication complex in the (−)-strand RNA genome, which is required for the subsequent (+)-strand RNA synthesis.

In this study we performed the head-to-tail ligation of RNA and qRT-PCR to detect and quantitate coronavirus (−)-strand DI RNA synthesis. The method, along with Northern blot assay, allows us to simultaneously detect both (−)- and (+)-strand RNA synthesis and specifically define the functions of *cis*-acting elements in the (−)- or (+)-strand DI RNA synthesis. In coronaviruses, besides the *cis*-acting elements identified here, many *cis*-acting elements located in the 5′ UTR, 3′ UTR and coding region have been demonstrated to be required for replication; however, their specific function in (−)- or (+)-strand RNA synthesis remains to be determined. Using the method may contribute to elucidate the specific roles of these *cis*-acting elements.

## Materials and Methods

### Viruses and cells

A DI RNA-free stock of the Mebus strain of BCoV (GenBank accession no. U00735) at 3×10^7^ PFU/ml was used as a helper virus in human rectal tumor (HRT)-18 cell line as described previously [56], [57], [58].

### Plasmid constructs

To generate the pBM25A construct, PCR was performed with the oligonucleotides TGEV 7(−) and BM25A(+) and with pDI RNA-1 in which the 288-nt 3′ UTR of BCoV-Mebus in pDrep1 was replaced with the 301-nt 3′ UTR and 43-nt poly(A) tail of MHV-A59 (GenBank accession no. NC_001846) as a template [26], [35]. The resultant PCR product was cloned into the TOPO-XL vector (Invitrogen) and digested with *Spe*I and *Mlu*I. The digested fragment was cloned into *Spe*I- and *Mlu*I-linearized pDI RNA-1 to create pBM25A containing MHV-A59 3′ UTR and 25-nt poly(A) tail.

To construct the mutant pΔ55, an overlap PCR mutagenesis procedure was performed as previously described [59] but with the oligonucleotides TGEV 7(−) and Δ55(+) and pBM25A DNA in the first PCR, the oligonucleotides Δ55(−) and DI reverse(+) and pBM25A DNA in the second PCR, and the oligonucleotides TGEV 7(−) and DI reverse(+) and the products of the first two reactions in a third PCR to make a 1203-nt product that was cloned into the TOPO XL vector (Invitrogen). From this, a 756-nt fragment obtained by digestion with *Spe*I and *Mlu*I was cloned into *Spe*I- and *Mlu*I-linearized pBM25A to make the mutant pΔ55. Mutants of pΔ1, pΔ2, pΔ3, pΔ4, pΔ5, pΔ15, pΔ30, pΔ55–45, pΔ55–40, pΔ55–35, pΔ55–30, pΔ30–15, pΔ5–14, pΔ15–24, pΔ25–34, pΔ35–39, pBM-3′A, pBM-3′G, and pBM-3′U were similarly constructed, except for the corresponding oligonucleotides used in the first and second reactions as described in Table S1.

### Head-to-tail ligation and RT-PCR for detecting (−)-strand DI RNA products

To synthesize transcripts *in vitro*, the *Mlu*I-linearized plasmid DNA construct was transcribed with the RiboMAX Large Scale RNA Production System-T7 (Promega) according to the manufacturer's instructions and was chromatographed through a Biospin 6 column (Bio-Rad) before use. To analyze the synthesis of the (–)-strand DI RNA (Fig. 2B), the HRT-18 cells in 35-mm dishes at ∼80% confluency (∼8×10^5^ cells/dish) were infected with BCoV at a multiplicity of infection of 5 PFU per cell. After 2 hours of infection, 3 µg of BM25A or mutant transcript quantitated by RNA denaturing gel was transfected into BCoV-infected HRT-18 cells. The total cellular RNA was extracted with TRIzol (Invitrogen) at 8 hpt, and 10 µg of extracted RNA in 25 µl of water, 3 µl of 10X buffer and 10 U of (in 1 µl) tobacco acid pyrophosphatase (TAP) (Epicentre) were used to remove the 5′-triphosphate groups of the (–)-strand DI RNA, generating monophosphate DI RNA (–) strand. Following the treatment of TAP, phenol-chloroform-extracted RNA in 25 µl of water was heat-denatured at 95°C for 5 min and then quickly cooled. Then, 3 µl of 10X ligase buffer and 2 U (in 2 µl) of T4 RNA ligase I (New England Biolabs) were added, and the mix was incubated for 16 h at 16°C. Phenol-chloroform-extracted ligated RNA was quantitated, and 1 µg of ligated RNA was used for RT reaction with oligonucleotide MHV3′UTR3(–), which binds nts 89–112 from the poly(U) tail in the (–) strand of the MHV-A59 3′ UTR, to synthesize cDNA with SuperScript III reverse transcriptase (Invitrogen). Of this, 5 µl was used in a 50-µl PCR with AccuPrime Tag DNA polymerase (Invitrogen) and oligonucleotide MHV3′UTR6(–), which binds nts 64-85 from the poly(U) tail in the (–) strand of the MHV-A59 3′ UTR, and BCV23-40(+), which binds nts 23–40 in the (+)-strand leader sequence of BCoV. The mixture was heated to 94°C for 2 min and then subjected to 50 cycles of 30 s at 94°C, 30 s at 60°C, and 30 s at 72°C. The ∼150- bp PCR product was sequenced after cloning into TOPO XL vector (Invitrogen).

### Quantitation analysis of (−)-strand DI RNA synthesis by qRT-PCR

For quantitating (–)-strand DI RNA synthesis, 1 µg of TAP-treated and ligated RNA was used for RT reaction with oligonucleotide MHV3′UTR3(–) and SuperScript III reverse transcriptase (Invitrogen). Real-time PCR amplification using the primers MHV3′UTR6(–) and BCV23-40(+) was performed using TagMan Universal PCR Master Mix (Applied Biosystems) according to the manufacturer recommendations with a LightCycler 480 instrument (Roche Applied Science). To quantitate the synthesis of (–)-strand DI RNA, dilutions of plasmids containing the same sequence as the detected RT-PCR product of (–) –strand DI RNA were always run in parallel with quantitated cDNA for use as standard curves (dilutions ranged from 10^8^ to 10 copies of each plasmid). In addition, 18S rRNA with primers 18S rRNA(–) and 18S rRNA(+), DI RNA with primers MHV3UTR2(–) and BCV29-54(+) and N sgmRNA with primers leader20(–) and BCVN(+) were applied as internal controls for normalization of (–)-strand DI RNA synthesis. The reactions were conducted with an initial pre-incubation at 95°C for 5 min, followed by 35 amplification cycles as follows: 95°C for 15 s and 60°C for 60 s.

### Northern blot assay for DI RNA replication

Ten micrograms of TRIzol-extracted total cellular RNA at 48 hpi of VP1 was used for electrophoresis in a formaldehyde-agarose gel. RNA was transferred from the gel to Nytran membrane by vacuum blotting, and the blots were probed with oligonucleotide TGEV 8(+) (for DI RNA), BCVN(+) (for N sgmRNA) or 18SrRNA(+) (for 18S rRNA), which was tailed with digoxigenin (DIG)-ddUTP using a DIG Oligonucleotide 3′-End Labeling kit (Roche Molecular Biochemicals). The RNA detected was visualized according to the manufacturer's recommended procedure.

### Sequence analysis of mutant DI RNA

To identify the sequence of the (–)-and (+)-strand DI RNA at 8 hpt and at 48 hpi of VP1, 10 µg of total cellular RNA was treated with tobacco acid pyrophosphatase (Epicentre) and ligated with T4 RNA ligase I (New England Biolabs). One microgram of ligated RNA was used for an RT reaction with oligonucleotide MHV 3′ UTR3(–) [for (–)-strand DI RNA] or BCV107(+) [for (+)-strand DI RNA] to synthesize cDNA with SuperScript III reverse transcriptase (Invitrogen), after which the oligonucleotides MHV 3′ UTR2(–) and BCV29-54(+) were used for PCR with AccuPrime Tag DNA polymerase (Invitrogen). The resultant PCR product was directly sequenced.

## Supporting Information

Figure S1 **Schematic diagram of the procedure to detect the synthesis of both (−)- and (+)-strand DI RNA.** The HRT-18 cells were infected with helper virus BCoV and then transfected with DI RNA. The virus contained within transfected cells was referred to as virus passage 0 (VP0). The total intracellular RNA was collected at 8 hpt to detect the synthesis of (−)-strand DI RNA with qRT-PCR. The supernatant collected at 48 hpt from the BCoV-infected DI RNA-transfected cells contained virus passage 1 (VP1) and was used to infect fresh HRT-18 cells. The total intracellular RNA collected at 48 hpi of VP1 was used to detect the synthesis of (+)-strand DI RNA with Northern blot assay. The 30-nt TGEV reporter is the binding site for the probe TGEV8(+) used in Northern blot assay.
(TIF)

Figure S2 **The synthesis of (+)-strand DI RNA as detected by Northern blot assay.** Total cellular RNA was extracted at 24 hpi of VP1 and was analyzed by Northern blot assay with N sgmRNA and 18S rRNA used as internal controls. The sequence of the BCoV DI RNA at 24 hpi of VP1 was determined by direct sequencing of RT-PCR products. wt: wild type, mut: mutant, mx: mixed.
(TIF)

Table S1 **Oligonucleotides used for this study.**
(TIF)
