# Supplementary figures and images for: The 3′-Terminal 55 Nucleotides of Bovine Coronavirus Defective Interfering RNA Harbor Cis-Acting Elements Required for Both Negative- and Positive-Strand RNA Synthesis
Source: PLoS One. 2014 May 22;9(5):e98422. doi: 10.1371/journal.pone.0098422 (PMC4031142; doi:10.1371/journal.pone.0098422)

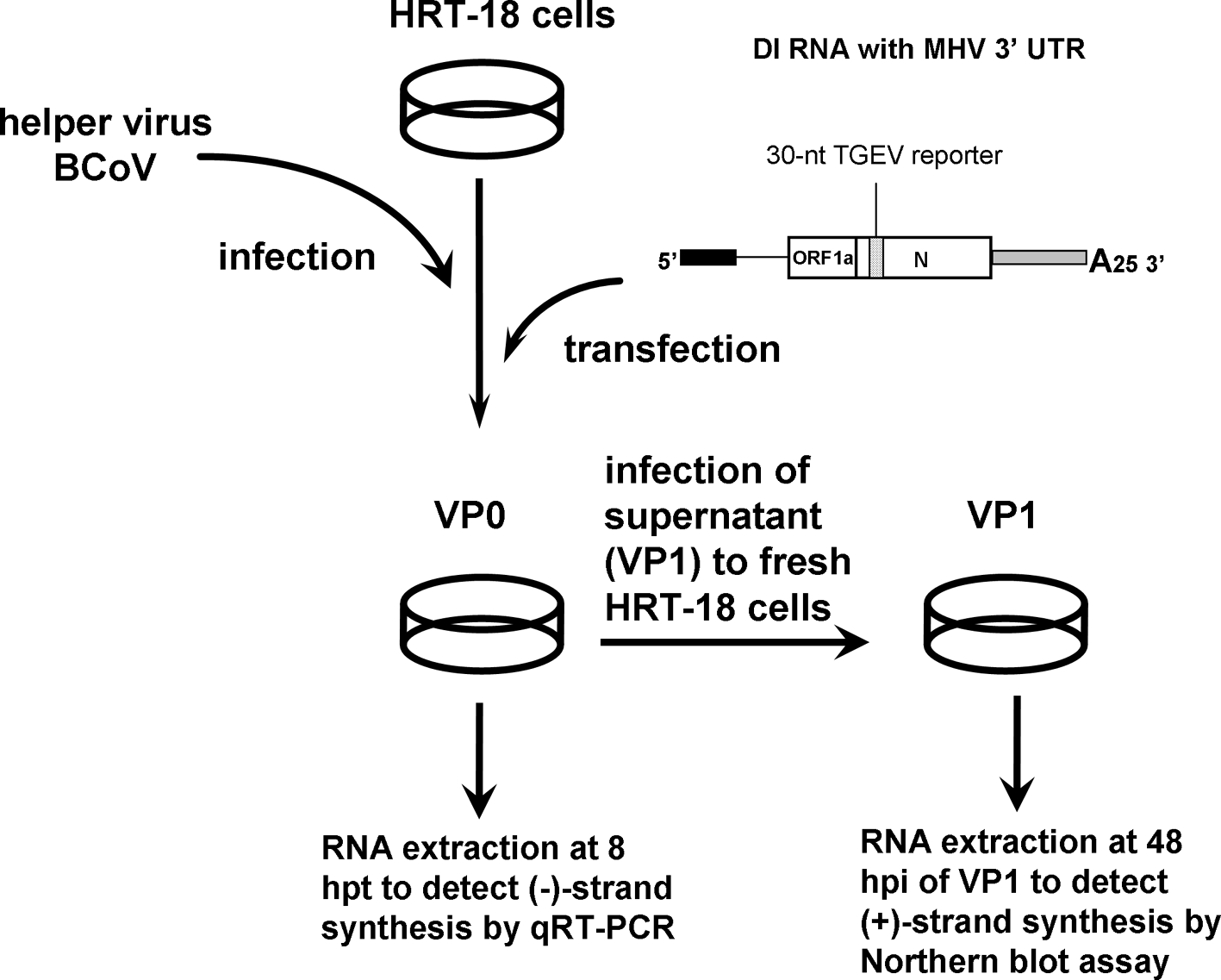

Supplement: Figure S1 — Schematic diagram of the procedure to detect the synthesis of both (−)- and (+)-strand DI RNA. The HRT-18 cells were infected with helper virus BCoV and then transfected with DI RNA. The virus contained within transfected cells was referred to as virus passage 0 (VP0). The total intracellular RNA was collected at 8 hpt to detect the synthesis of (−)-strand DI RNA with qRT-PCR. The supernatant collected at 48 hpt from the BCoV-infected DI RNA-transfected cells contained virus passage 1 (VP1) and was used to infect fresh HRT-18 cells. The total intracellular RNA collected at 48 hpi of VP1 was used to detect the synthesis of (+)-strand DI RNA with Northern blot assay. The 30-nt TGEV reporter is the binding site for the probe TGEV8(+) used in Northern blot assay. (TIF) [file pone.0098422.s001.tif]

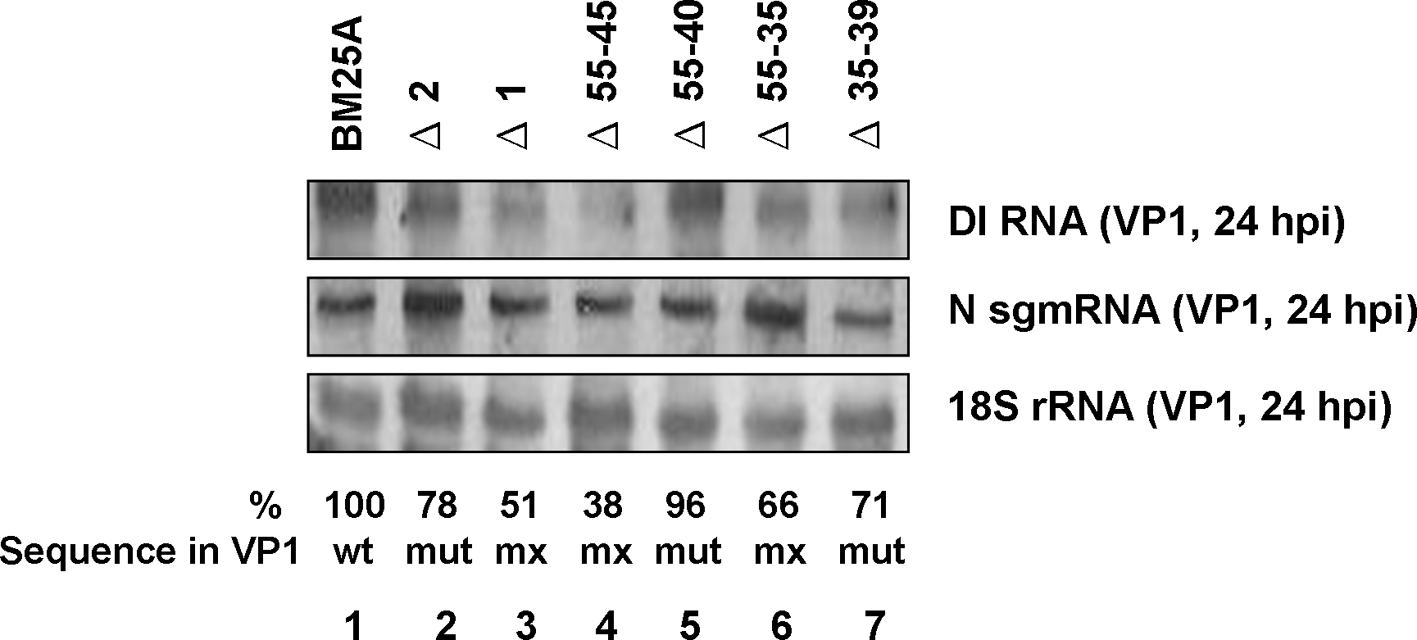

Supplement: Figure S2 — The synthesis of (+)-strand DI RNA as detected by Northern blot assay. Total cellular RNA was extracted at 24 hpi of VP1 and was analyzed by Northern blot assay with N sgmRNA and 18S rRNA used as internal controls. The sequence of the BCoV DI RNA at 24 hpi of VP1 was determined by direct sequencing of RT-PCR products. wt: wild type, mut: mutant, mx: mixed. (TIF) [file pone.0098422.s002.tif]

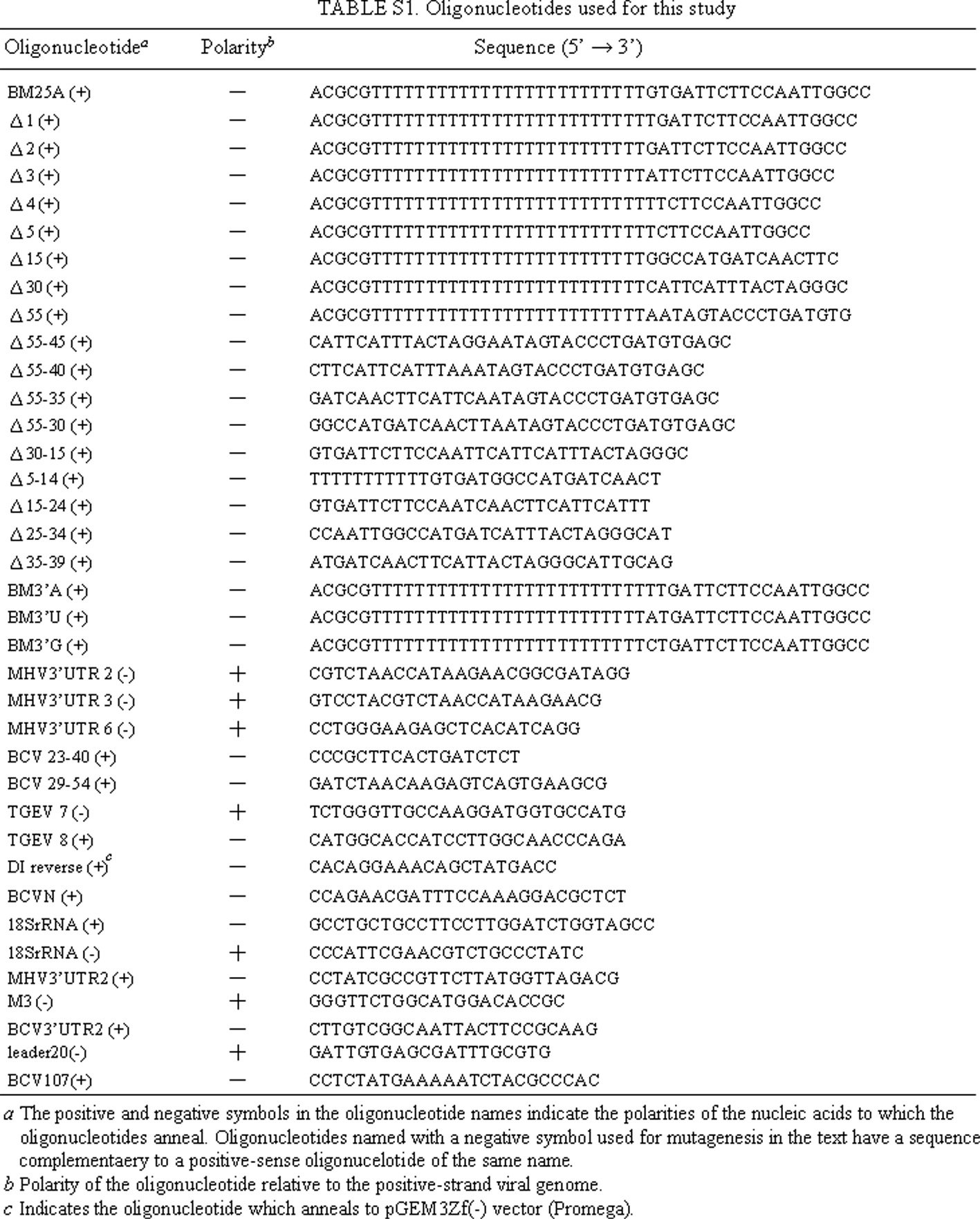

Supplement: Table S1 — Oligonucleotides used for this study. (TIF) [file pone.0098422.s003.tif]
